# Designing and analyzing the structure of Tat-BoNT/A(1-448) fusion protein: An *in silico *approach

**Published:** 2014-06

**Authors:** Jafar Amani, Parvaneh Saffarian, Shahin Najar-Peerayeh, Abbas Ali Imani- Fooladi

**Affiliations:** 1 Applied Microbiology Research Center, Baqiyatallah University of Medical Sciences, Tehran, Iran.; 2 Department of Bacteriology, Faculty of Medical Sciences, Tarbiat Modares University, Tehran, Iran.

**Keywords:** *In silico* analysis, Botulinum neurotoxin, Cell penetrating peptides, (CPPs), TAT peptide

## Abstract

*Clostridium botulinum *type A (BoNT/A) produces a neurotoxin recently found to be useful as an injectable drug for the treatment of abnormal muscle contractions. The catalytic domain of this toxin which is responsible for the main toxin activity is a zinc metalloprotease that inhibits the release of neurotransmitter mediators in neuromuscular junctions. A cell penetrating cationic peptide, Tat, which is a truncated N-terminal part of the Tat protein from human immunodeficiency virus, can help the toxin penetrate the skin uninvasively. This study aimed at an *in silico *analyses of the Tat-BoNT/A(1-448) fusion protein structure. A genomic construct was designed and optimized based on *E. coli *codon usage. The structure of mRNA as well as the properties of hypothetical chimeric protein was then analyzed by bioinformatic tools. Afterwards, the secondary and tertiary structures of the fusion protein were predicted by GOR4 and I-TASSER online web servers. The interaction with synaptosomal associated protein 25kDa (SNAP-25) was also analyzed as a natural substrate for the toxin. Based on the studied secondary and tertiary structures of the protein, the selected order of fusion proteins provides the natural activity of each peptide. Energy calculating data show that the acquired thermodynamic ensemble related to the mRNA structure was-1473.2 kJ/mol (-352.10 kcal/mol) and both total protein energy (Etotal) and shape related energy(Eshape) were calculated as -2294.2kJ/mol (-548.32 kcal/mol). The stability index of TAT-BoNT/A was computed to be 27.22 which has an acceptable stability as compared to that of native BoNT/A (22.39).

## INTRODUCTION

Botulinum neurotoxin type A (BoNT/A) is a product of anaerobic, gram positive bacteria, Clostridium botulinum type A [1, 2] which is constructed as a single chain 150 kDa polypeptide consisting of one heavy chain with binding and translocating domains (100 kDa) that bind to specific receptors on cholinergic neuron terminals and help the toxin to inter neuronal cells, and one light chain catalytic domain (50 kDa) responsible for the main activity of the toxin after entry [3]. The catalytic domain is a zinc metalloprotease and proteolysis which is a member of SNARE-complex (soluble N- ethylmaleimide-sensitive factor attachment protein receptor) called SNAP-25 peptide [3, 4]. This complex is responsible for the trafficking of vesicles containing neuro- transmitters in neuronal cells, hence inhibiting the release of vesicles containing a stimulatory neurotransmitter, acetylcholine, in neuromuscular junctions and temporary prevents normal muscle contractions by the botulinum toxin [3]. Recently, BoNT/A has been applied as an injectional drug in medical sciences in order to treat abnormal muscle contractions such as strabismus and blepharospasm and to reduce or remove face wrinkles [5, 6]. But injection has side effects including pain, redness, irritation, trauma and bleeding from the injection site [7, 8] It is, therefore, necessary to find noninvasive ways for the entry of this drug into skin cells. Among various methods used to transport the molecules into cells, the use of cell penetrating peptides (CPPs) is the most favorite [9]. Cell penetrating peptides have less than 30 amino acids and are cationic and often amphipathic peptides in physiologic conditions. They are capable of transporting various molecules (peptides, proteins and nucleic acids) to different kinds of cells and tissues with minimum toxicity. There are several types of CPPs used for transporting peptides into cells. TAT peptide(47-57), a truncated peptide derived from the Trans-activator of the transcription protein in human immunodeficiency virus, has been used frequently in protein transduction methods. TAT peptide(47-57), when fused with BoNT/A light chain may succor its penetration into skin in an uninvasive way. In order to draw the structural plan of such chimeric protein, online bioinformatics software was used. *In silico *based science provides a powerful tool for the analysis of structure stability, quantity of energy and protein functionality. In this study, results of an *in silico *analysis of TAT-BoNT/A protein linked by a hydrophobic linker are presented followed by a discussion of the data and information obtained through the study.

## MATERIALS AND METHODS

**Sequence analysis: **The amino acid sequence of botulinum toxin type A light chain (LC-BoNT/A) was retrieved from the online banks, Swiss-Prot and NCBI (A5HZZ9 [2-448], Botulinum neurotoxin type A, Clostridium botulinum, strain Hall/ATCC 3502) and was aligned and blasted.

**Construct design and optimization: **The amino acid sequence of LC-BoNT/A was fused to the amino acid sequence of TAT peptide(47-57) as a CPP. The two parts of the fusion protein were connected by means of a proper linker. In order to select the best CPP to carry BoNT/A, based on the highest structural stability and minimal structural changes, the secondary protein structure of the three most prominent CPPs (penetratin, transportan and TAT) with maximum efficiency were analyzed by GOR4 tool [10] when attached to LC-BoNT/A. The peptide causing less conformational changes in native BoNT/A structure was then selected (data not shown). Furthermore, several hydrophobic linkers were examined by the GOR4 tool [10] to separate two functional parts of the chimeric protein with or without minimal intrusion in their native protein secondary structure (data not shown). The resulting chimeric protein construct was back translated and optimized based on bacterial expression host, *E. coli *codon usage by java codon optimization tool (JCat) (http://www.jcat.de/), Optimizer web server [11-13] and gene script server (http://www.genscript.com/). GC percentage and codon adaptation index (CAI) were then calculated [14], and the nucleotide sequence of TaT-BoNT/A- light chain fusion protein gene was submitted to the NCBI gene bank (accession NO. KF445072).

**mRNA structure analysis: **The mRNA secondary structure of the recombinant protein was retrieved and its thermodynamic details were analyzed using the “mfold server” (http://mfold.rna.albany.edu/) [15, 16] and RNA fold web server (http://rna.tbi. univie.ac.at/cgi-bin/RNAfold.cgi).

**Chimeric protein properties: **Physiological properties of the chimeric protein were obtained using DNA star and PROTPARAM tools [17].

**Secondary and tertiary structure prediction: **Predictions of secondary and tertiary structures of the chimeric protein were made using GOR4[10], I-TASSER [18-

20] and Phyre version 0.2 (Protein Homology/analogY Recognition Engine) [21] online web servers respectively.

**Evaluation of structural modeling: **The resulting structural modeling was evaluated by RAMPAGE server [22] and DNA star tools. Also, the solubility of the recombinant protein was tested by PROSO online software (A sequence-based PROtein SOlubility evaluator) [23].

**Docking of chimeric protein with SNAP-25: **Since botulinum toxin is a proteolytic enzyme, the interaction of the designed recombinant protein with the natural substrate of BoNT/A, SNAP-25 protein was tested using Hex docking software, version6.12 (http://www.loria.fr/~ritchied/hex/) [24].

## RESULTS AND DISCUSSION

**Figure 1:**
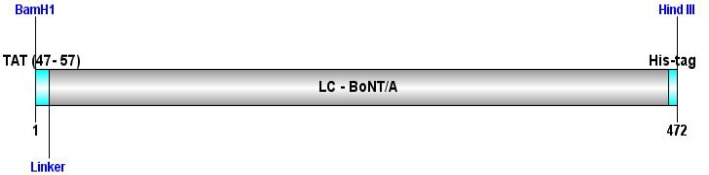
Sequence alignment of hypothetical chimeric protein. Schematic view related to amino acid sequence order of TAT-BoNT/A construct consists of TAT and BoNT/A genes bound together by appropriate linker for expression in E. coli. His- tag sequence was added to the end of sequence to ease detection of recombinant protein.

**Figure 2:**
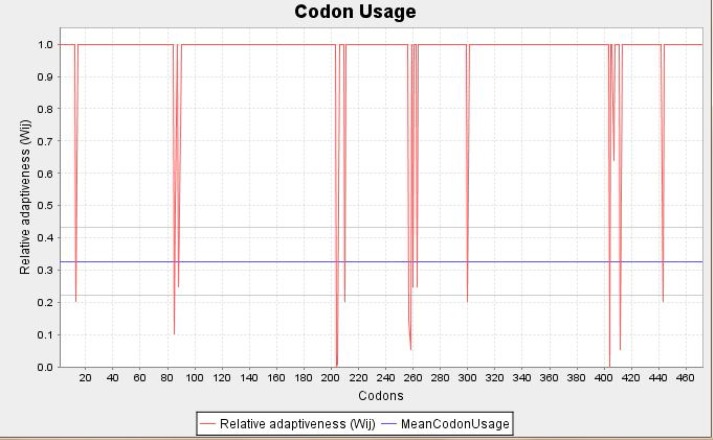
Adaptation of codon usage frequency distribution. Codon adaptation index (CAI) of 1.0 is considered to be perfect in the desired expression organism. The red line shows the Codon Usage for each codon present in the gene. The blue line depicts the mean Codon Usage in E. coli which was calculated for each known gene of this organism. The grey lines above and below the blue line mark the standard deviation for this mean codon usage in the E. coli.

**Sequence analysis and construct design**: The amino acid sequence of BoNT/A light chain (50 kDa) was retrieved from online gene banks and fused to TAT peptide(47-57) in a hypothetical genomic construct. On the other hand, several hydrophobic linkerswere examined to find the best linker to sustain functionality and retrieve the normal structure of the two parts of the recombinant protein (data not shown). Finally, GSGSGS sequence was selected as the linker to retain the flexibility of the construct. As shown in Figure 1, the *Eco*RI restriction enzyme sequence was inserted between two peptide sequences; hence we were able to separate the LC-BoNT/A later and to clone and express it as a negative control. Also, to ease recognition and purification, His tag sequence (6 His) was added to the C-terminal of the recombinant protein. Afterwards, the amino acid sequence (446 aa) was back translated and nucleic acid codons were optimized based on the codon labeled E.coli as the expression host. GC% codon usage bias in E.coli was increased by upgrading GC% and CAI to 46.18 (GC% of E.coli is about 50) and 0.92 respectively. CAI of > 0.8 was regarded as good, in terms of high gene expression level (Fig. 2).

**Figure 3:**
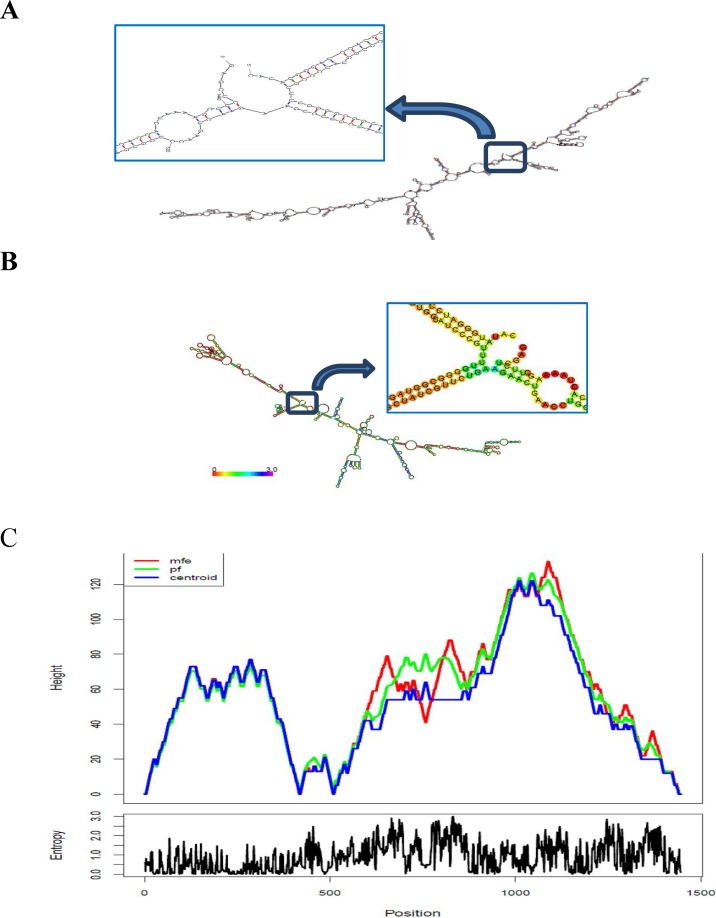
mRNA secondary structure prediction. **A)** The optimal secondary structure of TAT-BoNT/A mRNA. Predicted structure has no long stable hairpin and pseudo knot at 5′ site of mRNA. ΔG = -352.10. mfold tool. **B**) Minimal free energy (MFE) structure of TAT-BoNT/A mRNA drawing, encoding positional entropy. **C)** Prediction of mRNA secondary structure of TAT-BoNT/A chimeric gene. Centroid plain structure drawing related to TAT-BoNT/A mRNA structure

**mRNA structure analysis: **Secondary structure of TAT-BoNT/A mRNA was predicted using mfold [15, 16] and RNA fold web servers (http://rna.tbi.univie.ac.at/cgi- bin/RNAfold.cgi). Results are shown in Figure 3. The free energy of thermodynamic ensemble related to this structure is -1473.1864 kJ/mol (-352.10 kcal/mol). Other thermodynamic details are ΔH=-16904.1968 kJ/mol, ΔS=-49753.1992 kJ/mol and Tm =66.6°C assuming a 2 state model. Thermodynamic details related to 5’ end of TAT- BoNT/A mRNA were retrieved by mfold tool. According to Table 1, the minimum free energy (ΔG) of mRNA 5’ end is < -4 (-4.30) which does not constrain the hairpin loop structure.

**Table1:** Thermodynamic details related to 5’ end of TAT-BoNT/A mRNA. Minimum free energy (**ΔG**) of 5’ end is < -4 and it doesn’t constrain in hairpin loop structure

**Structural Element**	**ΔG**	**Information**
Helix	-4.30	3 base pairs
Hairpin loop Stack Stack Stack	4.50 -1.40 -1.40 -3.30	Closing pair is G456-C462 External closing pair is A4-U418 External closing pair is U5-G417 External closing pair is G6-C416
Stack Stack	-3.30 -2.40	External closing pair is G7-C415 External closing pair is G8-C414
Stack	-1.10	External closing pair is A9-U413

**Figure 4:**
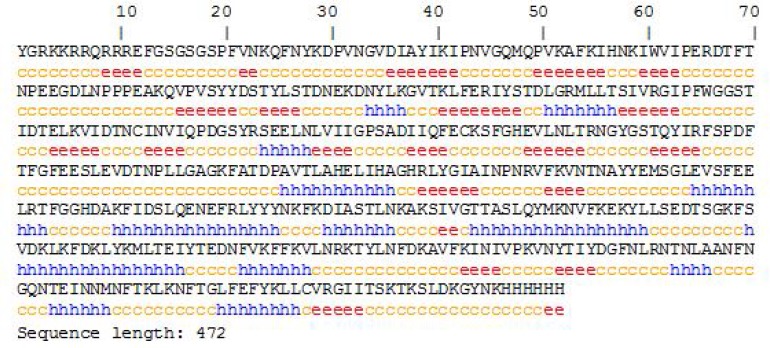
Protein secondary structure prediction. Secondary structure of TAT-BoNT/A chimeric protein, predicted by GOR4 tool

**Figure 5:**
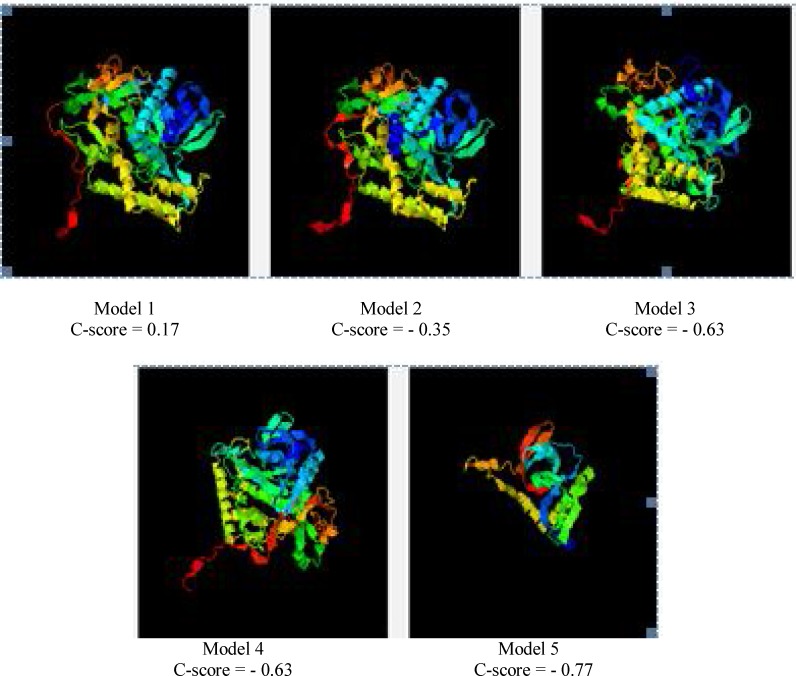
Tertiary structure prediction. Possible tertiary structures that were predicted by I- TASSER server. Based on C-scores, the model 1 has a high confidence between other four models.

**Chimeric protein structure prediction and evaluation: **The secondary and tertiary structure predictions of the chimeric protein were performed by GOR4[10], I- TASSER [20, 25] and Phyre v. 0.2 [21] online web servers, respectively. Ultimately, 5 possible tertiary structures were predicted by I-TASSER tool. According to C-scores calculated by this software, model 1, with a C- score of 0.17, had the highest confidence between the other four models. Data are shown in Figures 4 and 5.

Submitting the amino acid sequence of the recombinant protein in PROTPARAM web server revealed some of its physicochemical properties. For example, the number of amino acids was found to be 472 with a mo lecular weight of about 54 kDa. The isoelectric point was 9.02 with a net charge of about 10.83 at pH 7.0. Also, the total number of negatively (Asp + Glu) and positively (Arg + Lys) charged residues were 53 and 62 respectively. The estimated half-life of this recombinant protein was 2.8 hours (mammalian reticulocytes, in vitro), 10 min (yeast, in vivo) and 2 min (Escherichia coli, in vivo).

The instability index [26] of TAT- BoNT/A was computed by the PROTPARAM web server to be 27.22, which is smaller than 40, hence classifying the chimeric protein as stable. This is an acceptable stability as compared to the instabilit y index of native BoNT/A [22.39].

**Figure 6:**
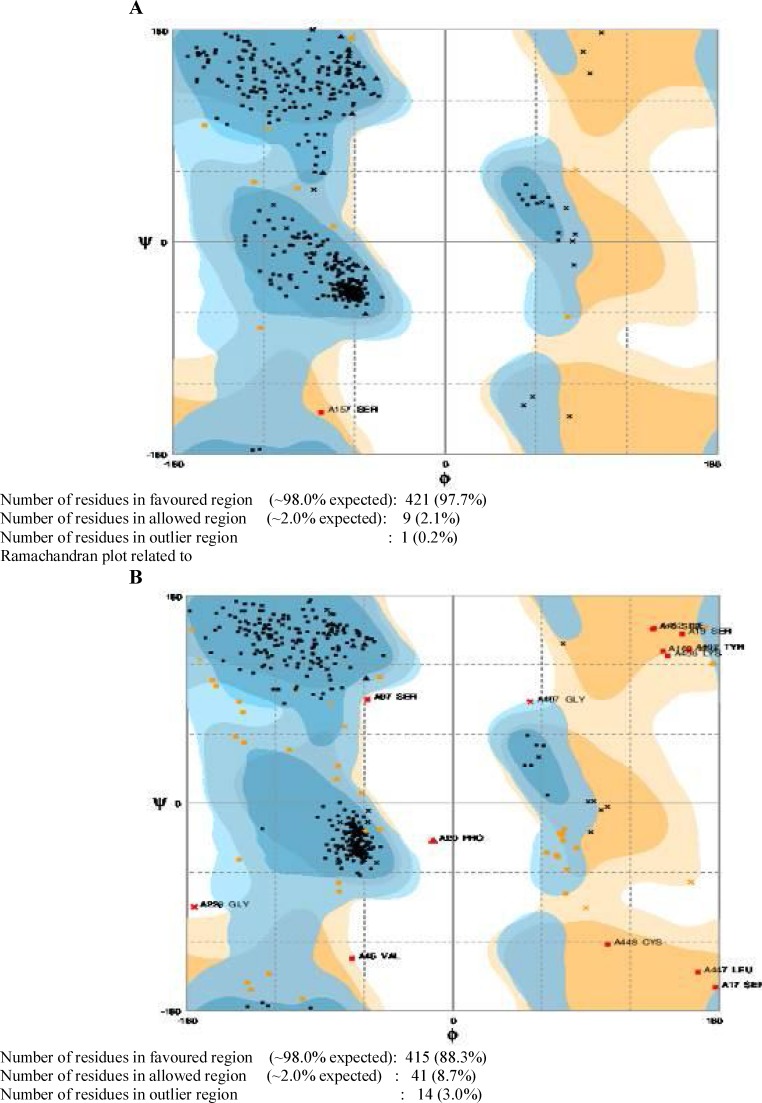
Ramachandran plot related to **A)** Light chain of BOT/A native protein and **B)** TAT- BoNT/A hypothetical designated protein

Moreover, a comparison was made between ramachandran plots related to the Light chain of BoNT/A native protein and TAT-BoNT/A hypothetical designated proteins using the RAMPAGE tool [22]. As evident from Figures 6A and 6B, the allowed φ, ψ backbone conformational regions in the two compared structures were very similar.

**Figure 7:**
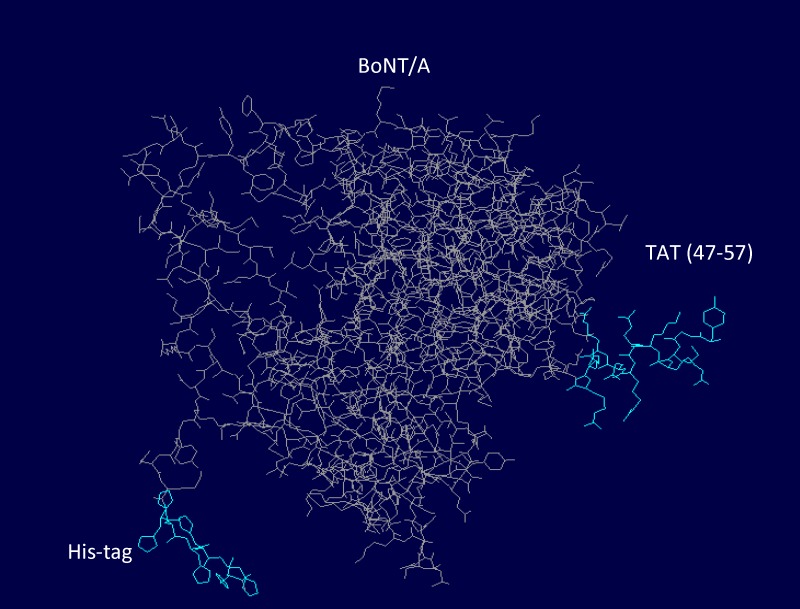
Three dimensional view of TAT-BoNT/A chimeric protein. Both TAT peptide(47-57)

The overall 3D view of the hypothetical recombinant protein designated in this study was structured by SwissPdb Viewer tool (http://www.expasy.org/spdbv/) [27]. As shown in Figure 7, the 3D model predicted this protein to be constructed of three separate parts containing BoNT/A, TAT peptide(47- 57) and His-tag (6 His). BoNT/A is the main part of the recombinant protein and is responsible for the main protein activity. The two other parts, TAT peptide(47- 57) and His-tag (6 His), are exposed in the surface of the chimeric protein which makes them reachable for receptor targeting.

and His-tag that are shown in image, made two separated domain.

To answer the question regarding the cloned proteins’ solubility chances for heterologous expression, we analyzed the amino acid sequence of the protein using PROSO online tool [23]. In this software, the input protein sequences are categorized into soluble and insoluble classes and a solubility score of 0-1 is provided. The calculated solubility of the recombinant protein in this study was found to be 0.631; with the score threshold value set to be 0.5 by default, hence classifying this recombinant protein as soluble.

**Figure 8:**
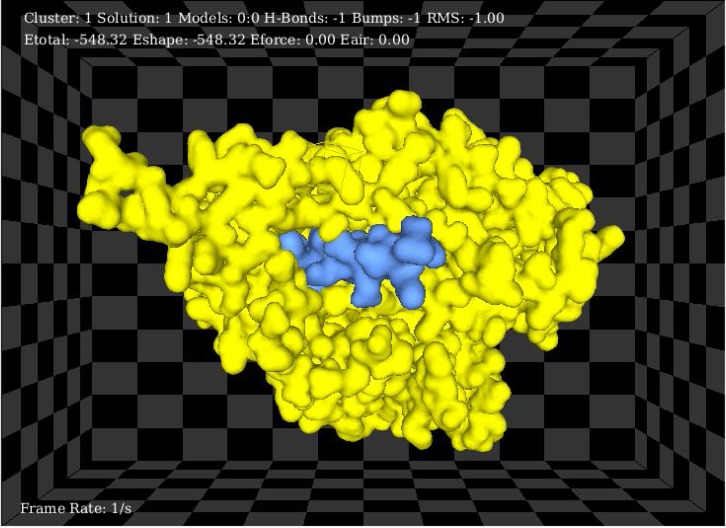
Protein-substrate interaction. Schematic view of docking model between TAT- BoNT/A and 17 residue of its ligand, SNAP-25 protein

**Docking chimeric protein with SNAP-25: **In order to predict the interaction of TAT-BoNT/A recombinant protein with its natural substrate, SNAP-25, a docking modeling was performed using Hex docking software, version 6.12 (http://www.loria.fr/~ritchied/hex/) [24]. Since recent documents have shown that C-terminal 17 residues of SNAP-25 are specifically required for interaction with BoNT/A [28], only this part of the SNAP-25 protein was used. The distance between TAT-BoNT/A and SNAP-25 protein was set on 30A˚ with a receptor/ligand range of 180˚ in 7.5 step size. According to the software analysis, total energy (Etotal) and shape related energy (Eshape) were calculated as -2294.17088 kJ/mol (-548.32 kcal/mol). The interaction of the enzyme- substrate is depicted in Figure 8.

Botulinum neurotoxin type A (BoNT/A) is composed of one functional domains; the catalytic domain (50 kDa) responsible for the main activity of the toxin, blocks the release of vesicles containing acetylcholine in synaptic junctions with zinc metalloprotease activity, followed by the inhibition of normal muscle contractions [6]. Recent studies show that due to the property of this very potential toxin, it can be used as an injectable biologic drug in the treatment of some muscle contraction disorders. To avoid side effects, researchers are looking for other less invasive ways. Many tools can help the direct delivery of drugs to the skin. Among them, the use of cell penetrating peptides (CPPs) is a more desirable method. To make BoNT/A permeable directly through the skin, we designed a gene construct containing BoNT/A catalytic domain fused to TAT(47-57), a cell penetrating peptide. In contrast to penetratin and transportan with high cellular uptake, TAT-fused protein cargoes show medium internalization into biological membranes [29]; on the other hand, peptides with high cellular entry decrease cell viability in the long run due to the formation of pores through cell internalization whereas TAT-mediated transduction shows no cytotoxicity even at high concentrations, because it occurs trough an endocytosis mechanism and does not disrupt biological cell membranes [29].

*In silico *science is an acceptable standard tool for gene designing and modeling

purposes and a very good substitute for time and money consuming experiments. Sometimes it is the only tool available to find best orders and conformations for desired gene constructs based on energy minimization and structural stability. Accordingly, our goal was to analyses a hypothetical chimeric protein by *in silico *modeling software.

In codon optimization, nearly all codons were shifted to the value 1.0 which is the maximum value. Codons considered as optimal were substituted by non-optimal codons in order to avoid undesired structures. Another reason for a "non-optimal" codon adaptation is avoiding cleavage sites of restriction enzymes. Also, a three dimensional model of the chimeric protein was analyzed using the I-TASSER tool; this software proposed five 3D model structures based on C-score which is a confidence score used for estimating the quality of predicted models (Table 2).

**Table 2:** Table score of different properties related to five TAT-BoNT/A tertiary structure models were predicted

**Name**	**C-score**	**Exp. ** **TM-score**	**Exp. ** **RMSD**	**No. of ** **decoys**	**Cluster ** **density**
Model 1	0.17	0.74±0.11	6.8±4.0Å	2027	0.2396
Model 2	-0.35			1213	0.1434
Model 3	-0.63			909	0.1074
Model 4	-0.63			916	0.1083
Model 5	-0.77			795	0.0940

It is calculated based on the significance of threading template alignments and the convergence parameters of the structure assembly simulations. C-score is typically in the range of -5-2, where a C-score of higher value signifies a model with a high confidence and vice-versa. Based on C-scores, model 1 has a higher confidence than the other four models. Analysis of the ramachandran plot related to the TAT-BoNT/A fusion protein by RAMPAGE software indicated that 415 (88.3%) residues were in the favored region (98.0% expected), as compared to native BoNT/A light chain with 372 (84.5%) residues in the favored region. Also, 41 residues (8.7%) of the TAT-BoNT/A fusion protein were in the allowed region, whereas, 54 (12.3%) residues in the LC- BoNT/A were in the allowed region. Moreover, three percent (14 residues) of each protein were posed in the outlier region [22]. With the help of *in vitro *data related to cationic peptides [30] along with information acquired from modeling studies, we propose that the TAT-BoNT/A(1-448) fusion proteins possibly retain their native conformation and therefore function for cell transduction and enzymatic actions inside the cells. Also, in theoretical models, CPP interacts selectively with polar heads of lipid bilayers. This property, along with polymorphism, is implicated in the mechanism of cargo-peptide cell transferring [31]. According to a study [32], covalent 1 to 1 cargo– CPP complexes could be more dependent on free CPP properties as compared to non- covalent CPP/cargo complexes, and are in immediate contact with the membrane, transferring through it directly.

Using online bioinformatics modeling tools, our data showed that the designated hypothetical chimeric protein has very good stability and solubility in presumptive environment conditions.
